# Targeting the ‘Undruggable’ Driver Protein, KRAS, in Epithelial Cancers: Current Perspective

**DOI:** 10.3390/cells12040631

**Published:** 2023-02-15

**Authors:** Kuen Kuen Lam, Siew Heng Wong, Peh Yean Cheah

**Affiliations:** 1 Department of Colorectal Surgery, Singapore General Hospital, Singapore 169856, Singapore; 2 J W BioSciences, Singapore 680226, Singapore; 3 Saw Swee Hock School of Public Health, National University of Singapore, Singapore 117549, Singapore; 4 Duke-NUS Medical School, National University of Singapore, Singapore 169857, Singapore

**Keywords:** KRAS, colorectal carcinomas, internal cancer drivers, small molecule inhibitor, macromolecular drug, adoptive cell therapy, bispecific antibody, antibody–drug conjugate, cytosolic antibodies

## Abstract

This review summarizes recent development in synthetic drugs and biologics targeting intracellular driver genes in epithelial cancers, focusing on KRAS, and provides a current perspective and potential leads for the field. Compared to biologics, small molecule inhibitors (SMIs) readily penetrate cells, thus being able to target intracellular proteins. However, SMIs frequently suffer from pleiotropic effects, off-target cytotoxicity and invariably elicit resistance. In contrast, biologics are much larger molecules limited by cellular entry, but if this is surmounted, they may have more specific effects and less therapy-induced resistance. Exciting breakthroughs in the past two years include engineering of non-covalent KRAS G12D-specific inhibitor, probody bispecific antibodies, drug–peptide conjugate as MHC-restricted neoantigen to prompt immune response by T-cells, and success in the adoptive cell therapy front in both breast and pancreatic cancers.

## 1. Introduction

Most (74%) of the genes strongly implicated in cancer by COSMIC encoded intracellular proteins which primarily localized to the cytosol or nucleoplasm. The U.S. Food and Drug Administration has approved drugs targeting human proteins from 618 genes, in which 535 proteins are targeted by synthetic small molecule inhibitors (SMIs) and only 108 are targeted by biologics. Most of these targets are predicted to be membrane proteins (59%), and 16% are secreted [1]. Thus, a substantial number of intracellular targets remain untargeted, which opens potential therapeutic opportunities.

Cancer is a major debilitating disease (https://seer.cancer.gov/statfacts/html/colorect.html) [2]. Colorectal cancer (CRC) is the third most frequent cancer and a leading cause of cancer mortality worldwide, attributable mainly to metastasis to distal organs such as liver and lung. Current chemotherapeutic drugs for advanced CRC have a dismayed success rate of less than 30% [3,4,5]. Despite better median survival in responders in the past two decades, respond rate to chemotherapy remained stagnant [6]. Patients who are not responsive to first-line chemotherapeutic drugs and whose KRAS status is wildtype are sometimes offered anti-epidermal growth factor receptor (anti-EGFR) therapy such as Cetuximab and Panitumumab. KRAS is downstream of EGFR, and hence patients who already have activating mutations in KRAS would not benefit from such therapy [7]. Nonetheless, the overall survival is only 4.6% to 12.3% for Cetuximab monotherapy and 6.9% to 18.9% for Cetuximab in combination with chemotherapy for these patients, who eventually also develop resistance due to the evolvement of mutations in KRAS [8,9]. Further, the majority (80%) of CRCs are microsatellite-stable and not responsive to immune checkpoint blockade therapy [10]. Hence, there is an urgent need to identify new therapeutics.

**Figure 1:**
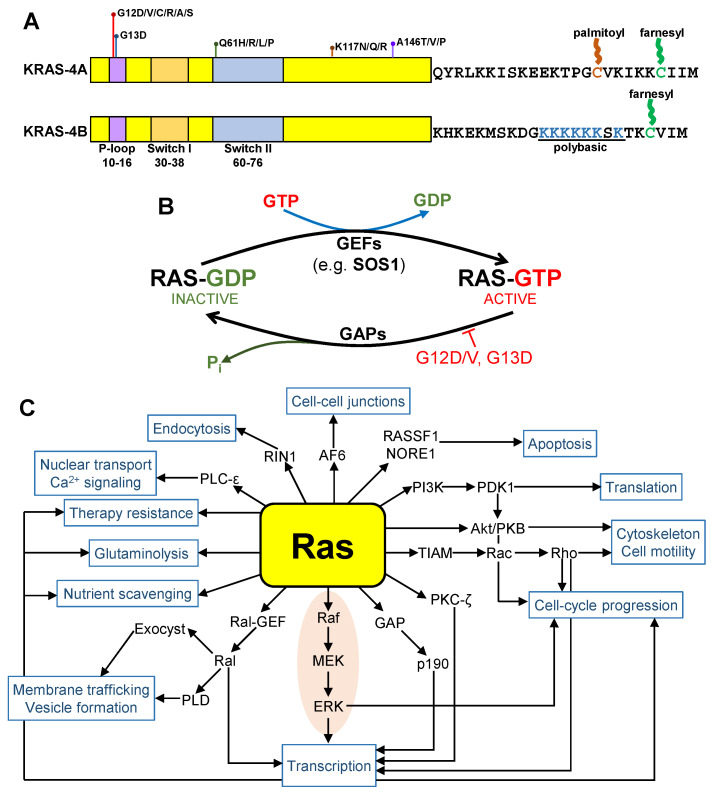
Protein domains of KRAS-4A and KRAS-4B. KRAS-4A and KRAS-4B differ in the C-terminal amino acid sequence (**A**). A simplified diagram of Ras GDP and GTP cycling into inactive and active states. (**B**) Simplified diagram showing the plethora of downstream pathways Ras activates (**C**) The RAF/MEK/ERK pathway is highlighted (orange background). Targeting the ERK and/or AKT pathways alone may not be sufficient.

KRAS is a gatekeeper gene in colorectal tumorigenesis as well as pancreatic ductal adenocarcinoma (PDAC) and non-small-cell lung cancer (NSCLC) and is thus an attractive target [11]. The KRAS 4B isoform is generally more abundant in CRC than the KRAS 4A isoform, although it varies from tumor to tumor (Figure 1A) [12]. KRAS is mutated in approximately 40% of CRC, more than 90% in PDAC and approximately 30% in NSCLC [11,13]. It is a small GTPase that shuttles between the inactive GDP-bound form to the active GTP-bound form [14,15]. The guanine nucleotide exchange factor (GEF) converts the GDP-bound KRAS to GTP-bound KRAS and the GTPase-activating proteins (GAP) reverse the process. When KRAS is mutated (mainly at residue 12 or 13), it is locked at the active mode as it no longer responds to GAP. Thus, KRAS-mutated cancers are actively signaling, resulting in uncontrolled cellular proliferation that eventually leads to carcinogenesis (Figure 1B).

In the present review, we compare and contrast various synthetic drugs and biologics for ‘drugging’ the KRAS molecule to illustrate the various methods developed to target intracellular diver genes for solid cancers and provide a current perspective.

## 2. Synthetic Drugs

The structure of the KRAS molecule is not easily amenable to inhibitor docking [16,17,18]. Efforts are diverted to downstream effectors such as the RAFMEK/ERK (also known as MAPK) and PI3K/AKT pathways [19,20]. Nevertheless, despite intense efforts for the past few decades, none of the inhibitors targeting ERK, BRAF, MEK or AKT have been translated into the clinic for CRC treatment. This is likely due to these molecules being the inappropriate target to inhibit KRAS signaling in human CRC [21]. Moreover, the authors showed that ERK1/2 are highly expressed in the matched colonic mucosa of the CRC patients; inhibiting these molecules may conceivably disrupt colonic homeostasis, which is thus not ideal. In addition, KRAS activates a plethora of pathways including metabolic scavenging (Figure 1C) [22,23]. Targeting downstream effectors to repress KRAS signaling may thus be less efficient and may elicit undesirable feedback loops.

### 2.1. Small Molecule Inhibitors (SMIs)

**Figure 2:**
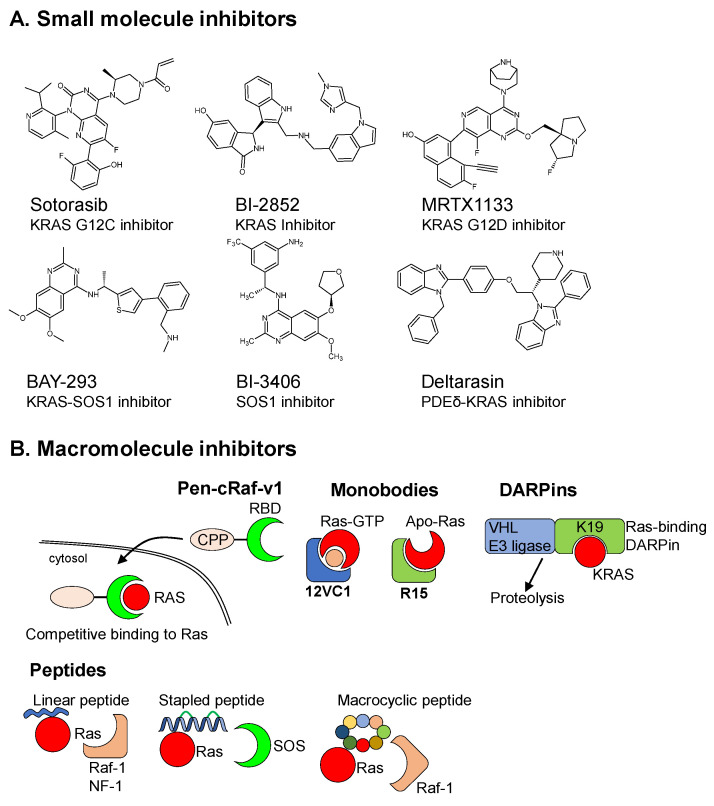
Key small molecule inhibitors (**A**) and macromolecule inhibitors (**B**) of Ras.

SMIs are drugs with molecular weights of less than 1kD that enable rapid penetration of cells. Recently, there has been a reverse trend to focus on KRAS itself, and a breakthrough has occurred in SMI targeting KRAS G12C mutation (Figure 2A). The discovery of Sotorasib (AMG 510) against KRAS (p.Gly12Cys) mutation in NSCLC has invigorated the race for other SMIs targeting KRAS molecule [24]. However, Sotorasib works by forming covalent bonds with cysteine (Cys). This might render reactivity of Sotorasib towards other cysteine-rich proteins which potentially affects its overall specificity and availability during chemotherapy. Furthermore, patients who initially responded to Sotorasib developed resistance within months, likely due to secondary mutation in other sites [25,26] and/or other diverse genomic and histologic mechanisms [27,28,29]. Other upcoming KRAS G12C inhibitors were extensively reviewed by Herdeis, et al. [30].

Nevertheless, KRAS G12C is a rare mutation in CRC. The majority of the KRAS missense mutations in CRC are G12D, G12V, and G13D [11,13]. A non-mutation-specific KRAS inhibitor BI-2852 (Figure 2A) that targets KRAS dimmer switch I/II pocket, which is the effector binding domain at nanomolar affinity, has shown promising results in downregulation of ERK in cell lines [31,32]. It has also showed some success in inhibiting cell proliferation in PDAC cell lines and patient derived 3D organoids [33]. No convincing in vivo results in animal models have yet been demonstrated. Furthermore, there is doubt as to whether this endpoint is applicable in human CRC as ERK1/2 expression in majority of human CRC tumors is significantly downregulated compared to matched mucosa [21].

More recently, advances in chemical technologies, increased knowledge of the KRAS Switch II pocket protein dynamics and high affinity interactions that increased non-covalent binding affinity by as much as 100,000-fold enabled the identification of a non-covalent KRAS G12D inhibitor, MRTX1133, that achieved drug-like potency [34]. This mutation-specific SMI (Figure 2A) showed promising anti-tumor activity in 2/5 and 1/5 patient-derived xenografts of PDAC and CRC tested, respectively [35]. The authors surmised that the different efficacy of the drug could have reflected the different biology of the cancers, PDAC being more homogenous than CRC. The authors also explored a combination of agents such as anti-*EGFR* and *PIK3CA* with MRTX1133 for the development of better therapeutic approaches.

Instead of targeting KRAS activation domain, efforts at the nucleotide exchange front have produced an inhibitor, BAY-293, that selectively inhibits KRAS-SOS1 interaction. SOS1 or son-of-sevenless is a GEF which catalyzes KRAS GDP exchange to GTP, rendering KRAS activation (Figure 1B and Figure 2A). BAY-293 completely inhibited downstream pERK activity in KRAS WT cells but only a ~50% inhibition in KRAS-mutant cells [36]. Plangger, et al. [37] showed that BAY-293 can work synergistically with glucose metabolism modulators, cell proliferation inhibitors, several chemotherapeutics and other modulators to exert cytotoxicity to Osimertinib-resistant NSCLC cells with mutated KRAS, and similarly for pancreatic cell lines [38]. BAY-293 has also been shown to suppress proliferation and colony formation of chronic myeloid leukemia cells with imatinib resistance and BCR-ABL independence [39]. A newer KRAS-SOS1 inhibitor BI-3406 has emerged, and the authors were able to show reduced KRAS-GTP complex in BI-3406-treated cells and hence less KRAS signaling despite the prevailing view that KRAS activating mutants are locked in the GTP-bound form [40]. BI-3406 enhances sensitivity of KRAS-dependent cancers to MEK inhibition by modulating feedback reactivation induced by MEK inhibitors. Both BAY-293 and BI-3406 were able to show antiproliferative potency in PDAC cell lines and patient-derived 3D organoids but were weakened by feedback regulation of the KRAS pathway [33]. Nevertheless, there are many GEFs (including SOS2) that can replace the function of SOS1; hence, it is unclear whether inhibiting SOS1 alone is sufficient to reduce KRAS signaling in humans to prevent cancer [41].

Mislocalization of KRAS from its site of function is another strategy to inhibit KRAS activity. KRAS activates its downstream effectors such as BRAF in the inner plasma membrane, and KRAS dwell time in the inner plasma membrane is correlated to its activity [42]. KRAS interaction with PDEδ is required for its inner plasma membrane localization [43,44]. Deltarasin (Figure 2A) is a high-affinity inhibitor of PDEδ-KRAS interaction and has been shown to kill lung and colorectal cancer cells [45,46,47,48,49]. Papke, et al. [46] and Martin-Gago, et al. [47] developed second generation PDEδ inhibitors with lower toxicity and higher selectively. However, PDEδ interacts not only with KRAS but also NRAS and HRAS, as well as several other farnesylated proteins, hence lacking specificity; it is not ideal as a clinical drug [50].

An emerging new class of SMIs called ‘molecular glue degrader’, such as the FL118 that target KRAS upstream regulator DDX5/p68, has promising results against mutant KRAS in vitro [51]. However, it is not apparent whether it is effective against any particular mutant KRAS. More recent evidence has indicated that inhibitors targeting related pathways are mutant-specific; for instance, the V-ATPase inhibitor is only effective against KRAS G12V and G13D mutants but not against G12D mutants [52].

There is also an ongoing debate on whether mutation-specific drug (such as Sotorasib, a KRAS(G12C) inhibitor) or pan-target drugs (such as BI-3406, a pan-RAS inhibitor) are optimal. The main advantage of the former is that it avoids wildtype reactivity [53] and is specific to a particular patient population. Hence, it may be more effective and less toxic whilst the latter may be more cost-effective from the public health perspective. For instance, KRAS is essential for adult hematopoiesis and hence inhibiting wildtype KRAS is undesirable [54]. The pros and cons of both strategies are succinctly summarized in a recent review [55].

In general, these SMIs targeting a particular functional domain of KRAS or other effectors of the pathway are effective specific intracellular therapy. However, patient response is usually not durable as resistance to these drugs invariably sets in rather quickly. Off-target toxicity is also a major problem [56].

### 2.2. Macromolecular Drug/Protein Scaffold

Macromolecular inhibitors usually have molecular weights of 5kD or more and hence need carrier to enter the cell. An area of focus is the screening for inhibitors against RAS effector binding domains to competitively inhibit binding of downstream effectors. Nomura, et al. [57] has identified a Pen-cRaf-v1 macromolecule (Figure 2B) with in vitro efficacy in inhibiting Ras activity comparable to BI-2852 [31]. Delivery of Pen-cRaf-v1 into cells is achieved by a cell permeable peptide (CPP) attached to its C-terminal. Nevertheless, Pen-cRaf-v1 suffers from poor in vivo efficacy due to poor blood stability.

Other creative methods include the use of protein scaffolds such as monobodies (Mb) (Figure 2B), for example, the 12VC1 Mb, which recognizes the active state of both KRAS(G12V) and KRAS(G12C) up to 400 times more tightly than wild-type KRAS [58]. The same research group also developed the R15 Mb against apo-RAS, the transient nucleotide-free RAS [59]. The authors showed that the Mb R15 is able to bind to apo-RAS and inhibit nucleotide exchange in KRAS wildtype and mutants that have high exchange rate (e.g., G12D, G13D). It is unclear whether the inhibition of such transient state is sufficient to inhibit KRAS signaling adequately in vivo. Moreover, these macromolecules enter the cell through endocytosis and hence face the same problem as antibody molecules (see discussion below).

Researchers have also resorted to using peptides to inhibit Ras as extensively reviewed by Pei, et al. [60]. Linear peptides [61,62,63,64], stapled peptides [65,66], and macrocyclic peptides [67,68,69,70,71] have been designed to inhibit KRAS (Figure 2B). Linear peptides mimic RAS binding motif from Raf-1 or NF-1 GTPase activating protein [61,62], or are peptide aptamers derived from two-bait two-hybrid system screening [63], or peptide dodecamer from phage library screening [64]. Stapled peptides are typically of α-helical structure constrained by a ‘staple’ which is a covalent linage between two amino acids of adjacent helices. Both Patgiri, et al. [65] and Leshchiner, et al. [66] designed stapled peptides based on SOS guanine exchange factor for Ras. Linear peptides face problems such as weak binding and susceptibility to proteolytic degradation while stapled peptides cannot be used to inhibit Ras-effector interactions which are not mediated by α-helices motifs. Macrocyclic peptides were therefore utilized as they can effectively inhibit protein–protein interaction. Wu, et al. [67] discovered a macrocyclic peptide which can inhibit Ras–Raf interaction from screening a one-bead-two-compound library against KRAS G12V. Similarly, Upadhyaya, et al. [68] discovered Cyclorasin 9A5 which can also block Ras–Raf interaction. Trinh, et al. [70] discovered a cell-permeable moderately potent macrocyclic peptide-based direct KRAS inhibitor, and Sakamoto, et al. [71] discovered KRpep-2d which inhibited enzyme activity of KRAS G12D with a 14-fold selectivity over KRAS WT. KRpep-2d suppressed ERK phosphorylation and A427 lung cancer cell proliferation. While peptides have shown promising in vitro results for direct KRAS inhibition, most are not cell-permeable, and thus they may be non-applicable as therapeutics.

Designed ankyrin repeat proteins (DARPins) are single domain engineered proteins (14 kDa) which are designed to bind to desired protein targets (Figure 2B) [72]. Bery, et al. [73] engineered the K19 DARPin peptide which specifically bind to KRAS encompassing His-95 but not HRAS and NRAS. They subsequently conjugated the Von Hippel–Lindau E3 ligase to the KRAS-binding K19 DARPin which tags the K19-KRAS protein complex for proteolysis. This is known as peptide–proteolysis-targeting chimeras or peptide-PROTAC [74]. The DARPin^®^ drug platform has been used to develop a few drugs which have made it to clinical trials though they do not act against Ras [75]. This is an exciting recent development. Nonetheless, the same limitation applies, i.e., the uptake of these macromolecules into human tumor cells in a specific, efficient and safe manner is yet to be demonstrated which may limit its application as therapeutics in humans.

## 3. Biologics

Biologics are products derived from living organisms by biotechnological manipulation. In the context of therapeutic targeting of KRAS, currently, two promising biologics are in the realm of T cell-based and antibody-based immunotherapy.

### 3.1. Adoptive Cell Therapy (ACT)

**Figure 3:**
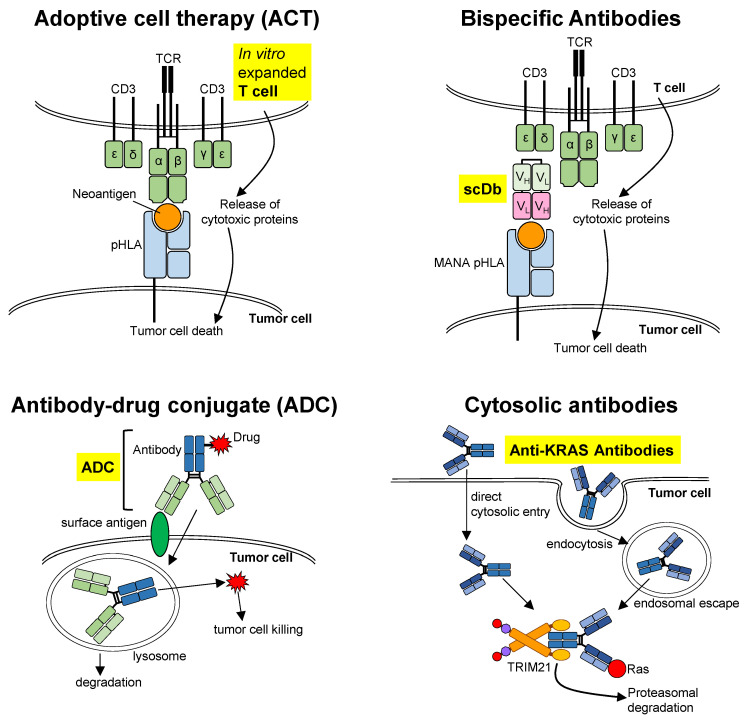
Biologics for Ras inhibition. Key components of the different approaches are highlighted (yellow).

ACT is cellular therapy by reinfusing in vitro manipulated and expanded autologous T-cells into pre-conditioned cancer patients (Figure 3) [53]. These T-cells are obtained either from Tumor-Infiltrating Lymphocytes (TIL) during surgery or from peripheral blood [76]. The patients undergo lymphodepletion before receiving the infusion and received a regimen of high-dose IL-2 to support the expansion of the infused T cells and hence patients often suffer from associated toxicities such as the cytokine release syndrome (CRS). Such therapy is highly dependent on specific T-cell receptors (TCR)—transduced T-cells, usually CD8^+^ effector memory T cells, and the presentation of neoepitopes by tumor-specific Human Leukocyte Antigen (HLA) molecules on the cell surface of tumor cells [77]. Chimeric Antigen Receptor (CAR)-modified T cells have limited success in solid tumor malignancies and will not be discussed here.

Two cases of successful ACT with public neoantigens from common KRAS G12D and p53 R175H mutations have demonstrated partial cancer regression for 6 months in a pancreatic and a breast cancer patient, respectively [78,79]. Nonetheless, most neoantigens are ‘private’ and hence ACT is a highly personalized therapy. One way to overcome this is to construct libraries of HLA-limited neopeptides of common mutation hot spots of gatekeeper genes such as KRAS and p53 [76]. Efficacy of ACT is dependent on retention of the TILs or TCR-transduced T cells over time and is possibly adversely affected by heterogeneity of HLA and antigen presentation mechanisms on the target tumor cells [78]. T-cell exhaustion could be another contributing factor limiting the success of ACT [80].

To overcome these limitations, researchers have made use of TILs to identify specific immunogenic mutations expressed on tumor cells to engineer tandem mini gene (TMG) mRNA vaccines for therapy [81]. Such vaccines could possibly improve ACT by restimulating T cells in vivo. Thus far, these lipid nanoparticles (LNP) encapsulated mRNA vaccines have been proven to be safe, but no clinical efficacy has yet been demonstrated.

To circumvent the toxicities associated with cellular therapy and to provide biologics that can reach more patients, researchers have developed bispecific antibodies to engage T-cells in killing tumor cells that displayed mutation-associated neoantigen (MANAs) on their surfaces.

### 3.2. Bispecific Antibodies

Bispecific antibodies are recombinant antibodies with two fragment antigen binding (Fab) regions, one targeting a receptor on T-cells, the other targeting an MHC-restricted antigen on tumor cells, thus enabling the recruitment of T-cells to kill tumor cells. CD3 bispecific antibodies (BsAbs) trigger CD3 receptor on Cytotoxic-T-Lymphocytes (CTL) and also bind to a second target on tumor cells (Figure 3) [82]. MANAs are a subclass of Tumor-Associated Antigens (TAA) contributed by hotspot mutations in cancer genes, e.g., KRAS, p53. Hsiue, et al. [83] and Douglass, et al. [84] described the development of CD3 BsAbs in the form of single-chain diabodies (scDbs), which recognize mutant TP53 and KRAS short peptides presented by HLA on tumor cell surface. These scDbs consist of a single-chain of T cell mimic antibody fragment (Fv) specific for MANA peptide HLA (pHLA), covalently linked to a scFv CD3 antibody which binds CD3 on T cells.

Since this method depends on HLA to present MANAs, one issue is frequent HLA downregulation in tumors [85] which possibly lead to low MANA pHLA presentation [82]. Douglass, et al. [84] showed that KRAS G12V 10-amino acid peptide is presented by HLA-A3 at only three and nine copies in lung papillary adenocarcinoma and pancreatic ductal adenoma carcinoma, respectively. Hsiue, et al. [83] showed the presence of only 2.4 copies of p53R175H/HLA-A2 per cell in a human multiple myeloma cell line. Nonetheless, both managed to show in vitro cancer cell killing despite low density of MANA pHLA presentation. Another problem is the highly polymorphic nature of HLA which increases difficulty of accurate epitope prediction. In vivo, scDbs suffers from instability to rapid clearance from blood in human and mice. Moreover, some solid tumors lack T cell infiltration which then rendered this method ineffective.

Probody therapeutic bispecific antibodies are masked antibodies designed to be masked in normal tissues but are activated in the tumor microenvironment by tumor-associated proteases [86]. Peptide masks to the binding domains of the target antigen and T cell are attached via protease-cleavable linkers. In the protease-rich tumor microenvironment, the linkers are cleaved, releasing the peptide masks, enabling binding of the Probody therapeutic candidate to the target antigen and CD3+T cells. Scientists from CytomX Therapeutics have designed Probody T cell-engaging bispecific (TCB) molecules for EGFR-CD3 and showed improved efficacy and reduced toxicity of this technology in preclinical (cell lines and cell line-derived xenografts) CRC models [87]. Although unmasked molecule could pose a safety concern, their data indicate that Probody TCB molecules remain masked in circulation and recirculation of cleaved molecule is minimal. It remains to be seen whether this works in the clinical context and whether appropriate cleavable mask to surface antigen of internal driver proteins such as KRAS can be designed and tested. Nevertheless, this technology suffers from the same limitation as other bispecific antibodies, that is, many patient tumors exhibit immunosuppressive tumor microenvironments with poor T-cell infiltration, which are expected to hinder activity.

Koide and Neel’s team has engineered hapten-peptide fragment encompassing *KRAS* G12C covalently modified by the inhibitor Sotorasib (Soto-p) based on the assumption that the drug-modified peptide could be a more effective MHC-presenting neoantigen than the naïve peptide generated by *KRAS* G12C. Soto-p is incorporated into the MHC complex (Soto-p/MHC) and used to successfully screen and identify a Fab clone R023 that has high affinity for Soto-p/MHC [88,89]. Engineered bivalent antibody of R023-scDbs comprising a Fab clone that recognizes the Soto-p/MHC complex on the tumor cells and a Fab that engages the T-cell receptor (TCR) CD3 complex expressed on T cells then elicit tumor cells killing by engaged T-cells. A critical feature of R023 scDb is that it only minimally recognizes free sotorasib or Soto-p, which enables coadministration of sotorasib and R023. This proof-of-concept study has successfully shown in vitro tumor cell killing. In vivo cell killing is yet to be demonstrated. This strategy nonetheless is dependent on efficient drug binding which could be weakened by second-site mutation.

In principle, bispecific antibody is a good strategy to kill neoantigen-presenting tumor cells by recruiting cytotoxic T-cells. However, this strategy may evoke immunotoxicity such as autoimmune response and is currently limited by stability of the scDb in the systemic circulation, the narrow spectrum of HLA alleles recognized and the paucity of T-cell infiltration in the tumor microenvironment of many epithelial cancers.

### 3.3. Antibody–Drug Conjugate (ADC)

ADCs are biopharmaceutical drugs that combine monoclonal antibodies (mAb) targeting surface antigens on tumor cells with anti-cancer SMIs linked by a chemical linker (Figure 3). This harnesses the specificity of antibodies to deliver the drug or payload precisely to the tumor cells and hence reduces systemic exposure. Normal cells which do not express the tumor surface antigen are spared and more toxic drug or higher dosage could therefore be deployed. Once internalized (via endocytosis or macropinocytosis, for instance), the antibody is degraded in the lysosome, releasing the payload to bind to the target, and causes cytotoxic death. In recent years, researchers have attempted to repurpose antibody, e.g., cetuximab (CTX) that is ineffective for KRAS-mutated cancers, to deliver highly toxic drug to treat KRAS-driven cancers such as PDAC.

The concept of ADC is simple, but the engineering faces numerous challenges. The site of coupling of the payload to the antibody may not always be easily controlled, which affects its safety and efficacy. Some researchers have devised unique conjugation methods such as by encapsulating the drug camptothexin in nanoparticle before conjugation with CTX [90] or re-bridging the inter-chain disulfides of CTX with the drug, auristatin-bearing pyridazinediones, to yield a highly refined ADC [91]. Another issue is the delivery of ADC to bulky tumors such as PDAC [92]. The ADC cannot penetrate the whole tumor to reach the inner tumor cells. Determining the pharmacokinetic and pharmacodynamic concentrations of both antibody and conjugate to determine in vivo activity is also very complex [91]. Though ADCs are touted to be tumor cell-specific, the highly toxic drug can also ‘leak’ to neighboring cells and cause bystander cytotoxicity, usually when cleavable linkers are used in the design, reaffirming the importance of the linker component in ADC. Unstable linkers can lead to premature release of the payload in the plasma, leading to neutropenia, thrombocytopenia, and hepatotoxicity [93]. The use of non-cleavable linker is an option; however, most payload requires conjugation via cleavable linker to achieve full potential. Nonetheless, linker is not the only component determining therapeutic window; the surface antigen, mAb structure, the degree of payload loading, payload type, and conjugation method can all impact ADC efficacy and toxicity profiles.

To date, no ADC delivering SMI targeting KRAS itself has been devised. In addition, ADCs cannot solve similar phenomenon faced by SMIs such as the reactivation of other effectors by negative feedback loop or therapy-induced resistance.

### 3.4. Cytosolic Antibodies

As the name implies, these biologics function by binding to intracellular driver proteins within the cytosol of tumor cells (Figure 3). Antibodies are attractive alternative therapeutics to SMIs as they are fully human in nature (and hence less toxic), have high affinity to their target proteins without having to bind to specific pocket, and have high serum stability and immune effector function. The design of SMI is dependent on tertiary structure of the target, whereas an antibody is easier to produce when structural data are absent [94]. Nevertheless, antibodies are much larger molecules (average size is approximately 150kD). Cellular entry is therefore a major limitation.

Antibodies which target self-antigens, also known as autoantibodies or autobodies, are detected in the serum of patients with autoimmune diseases such as Systemic Lupus Erythematosus (SLE). These autobodies have been reported to have the ability to penetrate cells to target intracellular antigens [95]. A review has highlighted the probability of direct membrane fusion of protein–nanoparticle supramolecular assemblies [96]. More evidence is provided by a recent review that some of these autobodies can enter cells via direct entry or via some other transporter mechanism such as the ENT2 nucleoside salvage pathway [97]. Direct entry of the antibody via endocytosis-independent pathway was also reported in a recent study (Lam et al., in submission).

Most antibodies nevertheless enter the cell via receptor-mediated endocytosis. Endosomal escape of the cargo into the cytoplasm which is highly inefficient and erratic [97,98] is thus a major limiting factor. Kim’s laboratory developed Cytotransmabs, which are intact, full-length IgG antibodies generated by incorporating a modified version of the cytosol-penetrating m3D8 VL moiety into the light chains (LCs) [99]. These are subsequently modified to produce pan-RAS Cytotransmabs, RT11, which blocks the localization of KRAS downstream effector eGFP-cRAFRBD (RBD: Ras-binding domain) fusion protein to the membrane in SW480 cells [100]. Further engineering produced a second generation pan-RAS monoclonal antibody inRAS37, which displayed improved endosomal escape efficiency of ~13–16% compared to ~4–6% of RT11, enhanced potency of antiproliferative effect on KRAS mutant cells and increased serum half-life in mice [101]. This antibody-engineering technology to enhance endosomal escape is remarkable. Nevertheless, the ways in which such engineering affects the stability of the antibody backbone and hence its function in vivo, and whether the high concentration of antibody used in these studies is achievable in humans to counter-cellular concentrations of Ras (estimated to be between 0.1–3.0 µM) are unclear [102]. Such engineering also affects the antibody Fc domain and hence TRIM21 binding site, thus abrogating the TRIM21-mediated proteosomal degradation process [103].

Other researchers have attempted to promote antibody cellular uptake via fusion of cell-penetrating peptides (CPPs) to C-terminus of the antibody, which does not affect the Fc domains. Zhang, et al. [94] developed 9D11-Tat recombinant antibody by fusion of HIV-derived CPP Tat to the C-terminal of 9D11 potent monoclonal antibody targeting intracellular Hepatitis B virus X (HBx). The recombinant 9D11-Tat antibody efficiently internalized into living cells via endocytosis and effectively inhibited viral transcription, replication, and protein production. Cytoplasmic HBx bound by 9D11-Tat was cleared by Fc receptor TRIM21-mediated intracellular neutralization [94]. Nevertheless, the authors did not report the endosomal escape efficiency or the target cell specificity of the antibody in this study. Subsequently, Gaston, et al. [104] reported that fusing CPPs to the C-terminus of the light chain of an anti-CEACAM5 antibody either before or after the hinge had the least effect on antibody developability and conferred target specificity. There was, however, no demonstration of functionality of the internalized antibody in the study. Of note, due to their inherent proclivity to interact with membranes, CPP sequences could interfere with the mammalian secretion pathway, which is undesirable [105,106].

Another approach to improve cellular uptake of the antibody without elaborate engineering of the antibody backbone is to encapsulate the antibodies in nanoparticles. Niu, et al. [107] have synthesized stable rough silica nanoparticles (RSNs) with controllable surface roughness for delivery of therapeutic anti-phospho-Akt antibody into breast cancer cell lines. They showed that the internalization of the antibody caused significant cell inhibition by blocking phospho-Akt and downstream anti-apoptotic Bcl-2 in cell lines. The use of silica-nanoparticles in biomedicine has been summarized in a recent review [108]. Others have devised strategy to enable direct cytosolic delivery of co-engineered protein-nanoparticles such as arginine-terminated gold particles via membrane fusion and showed cell killing in vitro [109]. Although inorganic nanoparticle–antibody delivery is promising, these carriers are toxic, and clearance from the human body may be problematic.

On the other hand, the safe and efficient implementation of the lipid nanoparticle (LNP)-encapsulated mRNA vaccine in the recent COVID-19 pandemic illuminates this as being an attractive alternative if it can be successfully adopted for antibody delivery. LNP comprises four lipid components: ionizable lipid that bind the cargo and shift the charge from positive to neutral once inside the cell to limit toxicity, helper lipids that aid in cell fusion, cholesterol that helps with endosomal escape, and polyethylene glycol-lipids that prevent particle clumping [110]. Tsourkas et al. [111] fused small protein scaffolds such as nanobodies or designed ankyrin repeats (Darpin) with anionic polypeptides (ApP). These small protein scaffolds were then encapsulated within cationic lipids or ionizable lipid nanoparticles for cellular delivery. They reported highly efficient cytosolic delivery (up to 90%) and inhibition of Ras and Myc function in two cell lines. The authors, however, did not report the endosomal escape frequency that could account for such high efficiency of cytosolic delivery. The use of these protein scaffolds (without the Fc domain of the whole antibody molecule) would also preclude the target protein complex from TRIM-21-mediated degradation and clearance. Although the authors have successfully used cationic lipids for encapsulation of whole antibody molecule [112], cationic lipids are toxic and unsuitable for delivery into humans [113]. It remains to be seen whether the whole monoclonal antibody can be as efficiently delivered into the cytosol via LNP encapsulation.

## 4. Conclusions

To target intracellular driver proteins, synthetic drugs, cellular therapy, and antibodies have been actively researched for several decades. The main limitation of SMIs is high off-target toxicity and low efficiencies due to feedback loops. Although this can be compensated somewhat by having combinatorial SMIs such as combining MEK inhibitor with AKT inhibitor, which do increase efficacy in some instances, toxicity is correspondingly increased [114,115,116,117]. In addition, detection of the best combinatorial drug strategy requires better comprehension of the SMI interaction, the unique mechanism of resistance, cancer type and its associated biology. This has been extensively discussed in a recent review [118]. Cellular therapy is a promising therapy, but it is also associated with high toxicity, personalized therapy and is perhaps suitable for terminally ill patients who have exhausted all avenues of treatment. In the future, co-culture of autologous TILs with senescent cancer cells may evoke a stronger antigen-dependent activation of CD8^+^ T cells and improve cellular therapy [119]. To deploy bispecific antibodies and ADCs to target intracellular driver proteins in humans, complex engineering designs are yet to be tested and validated to achieve plasma stability. These two methods have similar toxicity and therapy-induced resistance issues to those of SMIs. For cytosolic antibody to be efficient therapeutics against internal driver proteins, encapsulation/uptake method that ensures safe and efficient intracellular delivery without adversely affecting antibody function and efficient degradation or clearance of the target-antibody complex must be met. If these caveats are achievable, the cytosolic antibody platform could be a safer, less toxic and more efficient alternative to other methods discussed in this perspective for targeting internal cancer driver proteins. Moreover, if the mode of action is mis-localization rather than altered expression of the target protein, therapy-induced secondary mutations and resistance can conceivably be further delayed or reduced.

## Data Availability

Not applicable.
